# Effects of Supplementation of a Pre-workout on Power Maintenance in Lower Body and Upper Body Tasks in Women

**DOI:** 10.3390/jfmk4020018

**Published:** 2019-04-05

**Authors:** Michael Timothy Lane, Mark Travis Byrd, Zachary Bell, Tyler Hurley

**Affiliations:** 1 Exercise and Sports Science Department Eastern Kentucky University, Richmond, KY 40475, USA; 2 Kinesiology and Health Promotion, University of Kentucky, Lexington, KY 40509, USA; 3 Health, Exercise Science, and Recreation Management, University of Mississippi, University, MS 38677, USA

**Keywords:** pre-workout, power production, high-intensity cycle sprints, bench press

## Abstract

Currently there is a lack of research into how women respond to pre-workout supplementation. The effects of supplements on exercise performance in women, specifically to power, must be performed. This study investigated the effects of supplementation on power production and maintenance during a high-intensity cycle ergometry sprint performance, vertical jump performance, and bench press performance in women. It also investigated the effects of supplementation on power production and the maintenance of upper and lower body tasks in women. A total of 23 females (22.9 ± 3.6 years, 175.6 ± 6.5 cm, 86.9 ± 15.1 kg, 19.1 ± 8.4 body fat percentage (BF%) (mean ± std. dev.)) were familiarized with the testing protocol and maximal bench press performances were attained (49.5 ± 15.4 kg). Utilizing a double-blind crossover design, subjects completed three trials of: Five countermovement vertical jumps, a high-intensity cycle sprint protocol, which consisted of 10 maximal, five second cycle ergometer sprints. Subjects performed a velocity bench press test, utilizing 80% of their predetermined one repetition maximum (1RM) for 10 sets of three repetitions for maximal speed. For 20 min prior to each trial, the subjects ingested, in a randomized order, a pre-workout supplement (Supp), placebo+150 mg caffeine (Caff), or a placebo (PL). Peak power (PP), mean power (MP), and minimum power (MNP) were recorded for each sprint. Maximal velocity from each set was also recorded. Bike sprint and bench press data were normalized to the placebo trial for analysis. Blood lactate (bLa^−^) was measured immediately prior to each testing session, within 2 min of the completion of the last cycle sprint and following the bench press test. Bike sprint and bench press testing showed no significant differences through the testing sessions, but did significantly decline over test battery (*p* < 0.05). Vertical jump performance and lactate levels were not significantly different. Supplementation with a pre-workout supplement or placebo with caffeine 20 min prior to participation showed no positive benefits to performance in female participants.

## 1. Introduction

Pre-workout supplements (PWO) have become increasingly popular in gym culture and the military [1]. PWO are comprised of a variety of individual supplements to make a “proprietary blend”, which is formulated to have a synergistic effect to enhance performance [2]. Pre-workout supplements have shown to increase repetitions to failure in barbell movements and higher power production [3]. They have also shown to acutely improve choice reaction time, lower body muscular endurance, as well as improve perceived energy, alertness, focus, and reduce fatigue [4].

A number of individual supplements such as creatine, caffeine, and substances related to nitric oxide production are often selected as part of the pre-workout ingredients that have been widely investigated [2,5,6]. Creatine supplementation has shown to increase power output, muscle recovery, lean body mass, and delay fatigue in short duration high power exercises [7]. Caffeine supplementation has shown to cause a delay in fatigue, increase strength, and increase peak and mean power output [8]. Increases in nitric oxide production have been suggested to improve tolerance to physical activity by enhancing oxygen and nutrient delivery to the muscles being used during exercise [9]. Beet root extract and agmatine sulfate both contribute to nitrate and nitrite formation in the body, which in turn helps increase blood flow and has shown to have positive effects on performance [10,11,12,13]. Carnitine has been shown to increase time to exhaustion in running tests [14] and has been a component in other pre-workout supplements that enhance performance acutely [15]. Beta-alanine has been shown to have positive effects on exercise performance that lasts for 30 s to 2 min, however, these effects are typically observed with chronic supplementation (defined as consistent ingestion for ≥8 weeks) [2,16]. Overall, these compounds have been shown to have positive effects on acute single bout power performance and aerobic performance in individuals.

Improvements in cognitive performance have also been shown from pre-workout components other than caffeine. Choline bitartrate has shown to have an acutely positive effect on cognitive performance [17] while huperzine at 50 mg has shown to improve learning and memory performance [18]. Although these findings are promising, the positive effects have been primarily demonstrated in the animal model [19]. Overall pre-workout supplements have shown to be safe in female subjects when ingested chronically [20,21], but little research shows their effectiveness with increasing performance in women [22,23].

Research has shown how pre-workout, performance-enhancing supplements may improve peak power production [3] and how a pre-workout supplement affects maintained performance in males [5], however, previous studies have not examined how, or if, performance was maintained in females [3]. Peak power production has been tested by bike sprints, counter movement jumping, and barbell lifting performance, but research in this area has not thoroughly investigated the effects of this supplementation in women and often utilizes a mixed gender model [4]. Therefore, the purpose of this research study was to investigate the effects of supplementation on power production and maintenance during a high-intensity cycle ergometry sprint performance, vertical jump performance, and bench press performance in women. We hypothesized that the administration of a pre-workout supplement to recreationally trained females will increase performance in the upper and lower body.

## 2. Methods

### 2.1. Design and Participants

After approval from the institutional review board at Eastern Kentucky University and informed consent was obtained, 23 recreationally trained college-aged females (22.9 ± 3.6 years, 175.6 ± 6.5 cm, 86.9 ± 15.1 kg, 19.1 ± 8.4 body fat percentage (BF%) (mean ± std. dev.)) completed a double-blind crossover design study. Subjects had to be well versed in the barbell bench press and capable of performing maximal sprints on the cycle ergometer. Subjects were free of any major metabolic disease and did not have resting tachycardia (resting heart rate over 100 bpm), or high blood pressure (resting pressure ≥140/90 mmHg). A priori power analysis for this research study estimated a minimum sample size of 20 subjects with an alpha level set at 0.05, and a beta level set at 0.8 based upon pilot work data for changes in bench press velocity from supplementation taking average velocity performance from 0.5 to 0.55 meters per second.

There were three separate testing conditions (supplement—Supp, caffeine—Caff, and placebo—PL). The supplement was one serving of Muscle Pharm Ignite™ (Muscle Pharm, Denver, CO, USA) (Table 1), the placebo with caffeine was composed of 25 g of maltodextrin with 150 mg of caffeine flavored to match the supplement (the supplement contained 150 mg of caffeine), and the placebo was composed of 25 g of maltodextrin flavored to match the supplement. Each testing session lasted 60 min with each testing visit being set 5–9 days apart from the previous test. Subjects were instructed to abstain from exercise 24 h before each testing trial. Timing of subject testing was arranged so that subjects started the testing within 15 min of the first testing session to account for any circadian fluctuations in performance. Menstrual cycle timing was recorded during initial visits and times to avoid testing of subjects during menstruation. 

### 2.2. Protocol

#### Screening/Familiarization

After signing the informed consent form, the potential subjects were screened for general health and drug/supplement consumption by a questionnaire. Resting blood pressure and heart rates were recorded by utilizing a blood pressure cuff and taking a radial pulse for 15 s. Exclusion criteria from this study resulted if the subject reported any major health conditions (metabolic disease, cardiovascular disorders, etc.), had a high resting blood pressure ≥140/90 or heart rate HR ≥90bpm, chronically consumed supplements (taken at least 3 times per week), and/or had any orthopedic issues that would interfere with the exercise performance. Each subject was then provided a food log to self-report food intake for the 2 days prior to and the day of testing. Subjects were instructed to abstain from exercise and caffeine for 24 h before each testing session. Additionally, they were instructed to adhere to the same diet the day before each visit.

Following initial screening, the subjects completed a familiarization of the high intensity cycle protocol (5 bike sprints for 5 s each with a 55 second recovery), vertical jumps (2 repetitions), and bench press maximal strength test (1 repetition maximum (1RM)) (subject maximal strength results of 35.2 ± 9.6 kg) utilizing established strength testing methodology [17]. 

### 2.3. Testing

#### 2.3.1. Visual Analog Scale Testing

Upon arrival for each testing visit, subjects performed a visual analog scale (VAS) test set at a distance of 10 cm apart for energy, focus, fatigue, and anxiety/restlessness. The scales were counter weighted for low, or no being 1 and high being 10 for the energy and focus, the inverse for fatigue and anxiety/restlessness. These VAS tests were given directly before ingestion of the supplement condition, 20 min after ingestion of the supplement condition, after the cycle sprint battery and after the bench press testing. 

#### 2.3.2. Supplementation and Warm Up

The subjects ingested the randomly assigned treatment (Supp, Caff, or PL) by simple methodology at the start of each of the 3 testing visits. The subject’s blood lactate, utilizing a Lactate Plus Lactate Meter (Nova Biomedical Waltham, Waltham, MA, USA) was taken after ingestion, which would serve as the baseline measurement for the session. Twenty min after ingestion of the treatment (established from typical pre-workout supplementation studies [24,25]), the subjects completed a warm up on the cycle ergometer at a self-selected resistance for 5 min. Subjects then performed stretching as needed before starting the testing portion of the visit. 

#### 2.3.3. Vertical Jump Testing

Directly after warming up, subjects performed 5 maximal counter movement vertical jumps, reaching with their dominant hand on a Vertec (Power Systems, Boston, MA, USA). There was a 1-minute rest period between each jump. Subjects performed 5 additional maximal counter movement vertical jumps after completion of the cycle ergometer testing. Peak height was recorded overall for the pre- and post-cycle ergometer jumps.

#### 2.3.4. Cycle Ergometer Testing

After vertical jumping, subjects performed the sprint protocol on a Monark cycle ergometer (Monark Exercise AB, Vansbro, Sweden) model 894E, mechanically-braked cycle ergometer at 7.5% of their body weight applied as resistance (typical load utilized for Wingate testing). The testing protocol was pedaling against no resistance for 115 s followed by a resisted 5 second all-out sprint. Subjects then pedaled against no resistance, at a self-determined (50–70 rpm) rate for 55 s, followed by the next 5 second sprint. Subjects followed the 55 second rest and 5 second maximal sprint for a total of 10 sprints. After the sprints were all performed, subjects pedaled against no resistance for a 120 second cooldown. Blood lactate was measured 4–5 min following the completion of the last 5 second sprint. 

The following variables were recorded for each cycle testing trial: Maximum/peak power output (greatest power measurement produced during each sprint), mean power output (average power produced during each sprint), and minimum power output (lowest single power measurement produced during each sprint).

#### 2.3.5. Bench Press Power Testing

After the second round of vertical jump measurements, subjects performed a brief warm up on the barbell bench press with the empty barbell (20.4 kg) for 10 repetitions followed by 50% for 5 repetitions and then 70% of their 1RM for 3 repetitions. Subjects then performed 10 sets of 3 maximal velocity repetitions at 80% of their previously attained 1RM (to the nearest 2.4 kg increment). After the bar was returned to the rack for each set, a 1 minute rest period was started. All repetitions were measured for (peak and mean) velocity and power utilizing a linear position transducer (Gymaware, Sydney Australia).

Subjects were given one final VAS and a final blood lactate measurement. Subjects then scheduled their next testing visit. Each subject performed the same testing protocol, during each of the 3 testing visits with the only difference being treatment ingested.

#### 2.3.6. Statistical Analyses

All data collected was entered into Microsoft Excel 2016 (Microsoft Corp, Redman, WA) and then imported into the Statistical Package for the Social Sciences (SPSS) version 23 (IBM, Armonk, NY, USA) for analysis. Descriptive statistics were reported for each variable. VAS and lactate data was normalized to the first VAS metric and lactate score of the session. Data was analyzed by repeated measures analysis of variance (RMANOVA) with least squared difference (LSD) post hoc analysis (for treatment) through the course of the experimental visits. Statistical significance was set with an alpha level of *p* ≤ 0.05. Physical performance data was analyzed for best performance in each session for analysis. All physical performance data from the trials was then normalized to the placebo visit. Cycle sprint mean power performance, vertical jump height, and bench press velocity metrics were analyzed by treatment utilizing an analysis of variance (ANOVA) with least square differences (LSD) post hoc analysis. 

## 3. Results

### 3.1. Familiarization Data

Preliminary testing of the subjects gave a bench press 1RM of 35.2 ± 9.6 kg, cycle sprints peak power performance of 7.77 ± 1.45 W/kg, mean power performance of 7.04 ± 1.43 W/kg, and power drop performance of 1.72 ± 0.76 W/kg. Vertical jump performance during the first session was 48.9 ± 13.5 cm, with an average standing reach of 210.8 ± 8.4 cm. 

### 3.2. VAS (Visual Analog Scale)

#### 3.2.1. Energy

Over the testing session, the initial pre-treatment VAS score for energy was 5.38 ± 1.52. For the post treatment VAS score for energy was 6.23 ± 1.36. The post cycle sprints VAS score for energy was 5.38 ± 1.60. The post bench press VAS score energy was 5.65 ± 1.44. Utilizing an ANOVA with LSD post hoc analysis for treatment there was a significant time effect with greater energy pre-cycle sprints and lower energy ratings post-cycle sprints (*p* < 0.01). However, there were no significant differences between the treatment groups (Supp, Caff, or PL). 

#### 3.2.2. Focus

Over the entire testing session the pre-treatment VAS score for focus was 5.8 ± 1.55. For the post-treatment VAS score for focus was 6.27 ± 1.48. The post-cycle sprints VAS score for focus was 5.97 ± 1.60. Finally, the post bench press VAS score for focus was 6.05 ± 1.54. Utilizing a RMANOVA with LSD post hoc analysis for treatment there was a significant time effect with greater focus pre-cycle sprints (*p* < 0.05). However, there were no significant differences between the treatment groups.

#### 3.2.3. Fatigue

Over the entire testing session the pre-treatment VAS score for fatigue was 3.95 ± 1.96. For the post supplementation VAS score for fatigue was 3.42 ± 1.96. The post-cycle sprints VAS score for fatigue was 4.60 ± 1.77. The post bench press VAS score for fatigue was 4.05 ± 1.26. Utilizing a RMANOVA with LSD post hoc analysis for treatment there was a significant time effect with less fatigue pre cycle sprints and more fatigue post cycle sprints and bench press testing (*p* < 0.05). However, there was no significant difference between the treatment groups.

#### 3.2.4. Anxiety

Over the different treatment sessions the average performances were as follows: The pre-treatment VAS score for anxiety was 3.38 ± 2.10. For the post-treatment VAS score for anxiety was 3.39 ± 2.03. The post cycle sprints VAS score for anxiety was 3.06 ± 1.91. The post bench press VAS score for anxiety was 3.06 ± 2.00. Utilizing a RMANOVA with LSD post hoc analysis for treatment there was no significant differences between the treatment groups, but there was a significant decrease in anxiety after the cycle sprints and bench press testing (*p* < 0.05).

### 3.3. Vertical Jump

Of the 5 jumps performed, the maximal height was utilized for analysis. Pre-bike sprints, vertical jump height was 51.5 ± 6.9 cm and post-vertical jump was 51.5 ± 7.1 cm.

Vertical jump performance was analyzed utilizing ANOVA with LSD post hoc testing for difference between the treatment groups and vertical jump height between either pre- or post-cycle sprints performance found no significant differences. There were no significant differences in vertical jump performance pre to post sprinting.

### 3.4. Lactate

**Figure 1:**
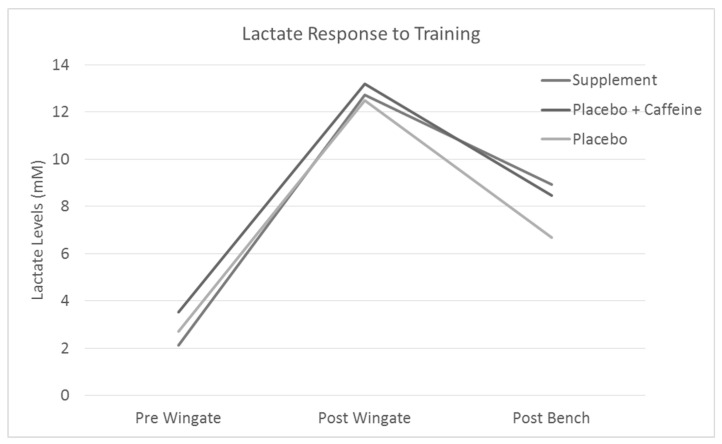
Graph for lactate levels at each of the three time points with lines for each different treatment trial average.

Pre cycle sprints lactate levels were 2.77 ± 1.61 mM. Post-cycle sprints blood lactate levels were 10.95 ± 3.28 mM. Finally, post bench press lactate levels were 7.38 ± 3.62 mM. Overall, there were significant differences in lactate levels for each time point (*p* < 0.05) (Figure 1), and utilizing LSD post hoc testing by treatment found no significant differences between the different treatments.

### 3.5. Cycle Sprints

**Figure 2:**
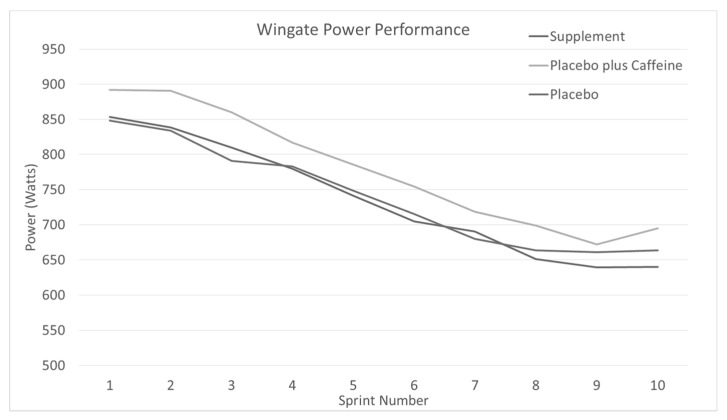
Bike sprint peak power performance across the sprints with different color lines for each treatment.

Individual sprint performance mean power relative to body mass output across each treatment is listed in Table 2 and Figure 2.

The sprints peak, mean, and minimum power significantly decreased across the trials (*p* < 0.01). There were no significant differences between any treatment at any time point or with best performances.

### 3.6. Bench Press

**Figure 3:**
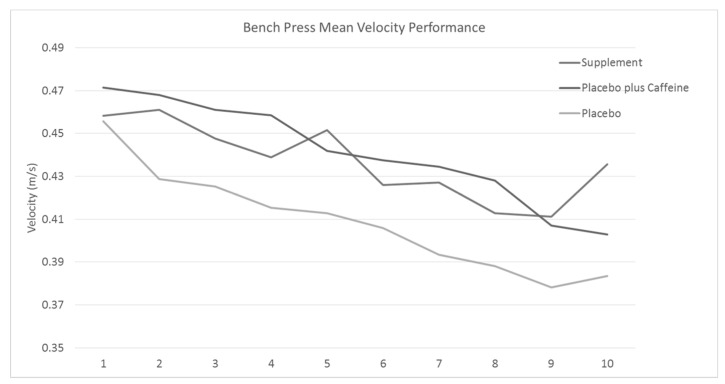
Mean velocity performance for the bench press across each of the 10 sets by treatment.

The bench press maximum for the participants was 35.2 ± 9.62 kg. Mean subject performance for average and peak velocity is expressed in Figure 3 and Table 3. Peak velocity average across the 10 trials was 0.665 ± 0.026 m/s supplement, 0.620 ± 0.025 m/s caffeine, and 0.624 ± 0.024 m/s for the placebo group. Mean velocity average across the 10 trials was 0.47 ± 0.019 m/s supplement, 0.458 ± 0.02 m/s caffeine, and 0.443 ± 0.018 m/s for the placebo group. Bench press peak velocity performance did decrease over the course of the bench press trials overall (*p* > 0.01) but there were no significant difference between the treatment groups. 

## 4. Discussion

Research that has examined the effects of comparable pre-workout supplements in men have seen both positive impacts on performance [5,19,25], and no impact [26]. Similarly, this study did not find any significant effect on power performance increases over the length of a high intensity sprint battery. The lack of effect could be caused by differences in testing duration, gender, or caffeine dosage [25]. Research studies where subjects performed sets to fatigue or longer testing protocols that are aerobic in nature have shown an effect from pre-workout supplementation [19]. Currently there is little available research looking into the effects of pre-workout supplementation and its effects on female exercise performance, whereas there are multiple studies showing the effectiveness in men [5,26]. Finally, research has shown the optimal amount of caffeine to supplement for athletic performance seems to be between 3–6 mg/kg of body mass and this supplement would have been on average for our subjects a dosage of approximately 2 mg/kg [27]. 

One of the individual ingredients within the Ignite pre-workout, beta alanine, has been shown to improve jump performance through an improvement in power performance of the same style of jumping used in this study [28]. The results from the tested jumping did not show the same response, which could be due to the gender difference, dosage, and/or lack of chronic usage. Greater amounts of caffeine and beta alanine could perhaps have increased performance on the vertical jumping since this has been observed in previous research [26,27]. Chronic consumption of beta alanine has been shown to increase peak power and would quite possibly have a positive effect on this performance, but an individual dose will not have those effects [24].

Blood lactate levels elevated significantly from the bike sprints, however, treatment had no additional effect on response. This was expected since the amount of work performed (and the intensity of it) was the same for all trials and hard anaerobic work has been shown to increase blood lactate levels [29]. There was no ingredient in the pre-workout supplement that would suggest there would be a decrease in lactate response to exercise. Changes in lactate response are typically observed due to the long term effects of training [29].

Data from the VAS did show significant effects of testing in that subjects rated higher levels of fatigue and lower anxiety and energy after the cycle ergometer test and bench press protocol. Furthermore, focus was highest before the cycle ergometer testing over the other testing sessions. There was, however, no significant difference in any time point due to supplementation. This is interesting, as previous research in examining the effects of perceived energy, focus, and fatigue in combination with the use of a pre-workout type supplement was shown to be significantly higher with the use of supplements (specifically caffeine), compared to placebo [27]. Other research has attributed this increase in perceived energy, focus, and decrease perceived fatigue to be possibly be caused by the caffeine within the pre-workout [18], but the results obtained point to the contrary. This is again likely due to the lower overall dosage of the caffeine given to the subjects as opposed to the higher dosage typically observed in the literature [27]. 

Further research is needed on women, specifically for timing and dosage for pre-workout supplementation. Dosing with a serving as opposed to a body weight relative amount was shown in this study to not give a significant result to most subjects. Typical caffeine supplementation research has shown the use to be effective at dosages of 3–6 mg/kg of body mass and the dosage level in the supplement in this study would have required subjects to have a body mass of 50 kg or less in order to be effective and our subject’s average body mass was 63 kg [28]. Research into the chronic effects of this supplementation must also be investigated to show overall, how long-term usage can affect performance both acutely and chronically. 

There were a number of limitations in this study. The subjects we tested were moderately trained, having a group of highly trained or completely novice individuals might have shown greater effects from supplementation [27]. The amount of sleep and recovery that the subjects had before each visit was not controlled and this could have influenced performance on their testing visits [30]. Subjects were told to abstain from hard training, but were not monitored directly on this point nor was their diet, which could both influence their subsequent visit performance. The load on the bench press and on the bike sprint were shown to induce fatigue in pilot work, but perhaps a lighter load for a greater duration (volume) would allow for greater changes in performance over the testing battery specifically by treatment [25]. 

## 5. Conclusions

Overall supplementation had no effect on performance when compared to a placebo or placebo with matched caffeine consumption. There are a number of potential reasons for this, such as the protocol was too intense and no supplementation would help mediate that decline, and that the dosage was perhaps too low and a greater amount of the supplement would be needed to show ergogenic effects. Further research must be conducted, utilizing chronic loading as a number of the ingredients were shown to have chronic effects from use and not acute effects.

## Figures and Tables

**Table 1:** Ignite supplement nutrition facts.

Supplement Facts		
	Amount Per Serving	%DV
Calories	5	
Total Carbohydrates	1 g	<1%
Sugars	0 g	
FitMiss Ignite Blend	5.85 g	
Carnosyn Beta Alanine, Choline Bitartrate, l-Tyrosine, l-Glycine, Taurine, l-Carnitine, Betaine Anhydrous, Hawthorn (Crataegus Pinnatifida) Berry, Agmatine Sulfate, Caffeine Anhydrous, Huperzine A 1% (Huperzia Serrata)		
Other Ingredients: Natural & Artificial Flavors, Ctric Acid, Malic Acid, Calcium Silicate, Sucralose, Silicon Dioxide, Acesulfame Potassium, Fruit & Vegetable Juice (Color).		

**Table 2:** Individual sprint performance mean power relative to body mass output across each treatment.

Round	Supplement	Caffeine	Placebo	Overall
1	8.63 ± 1.32	8.55 ± 1.04	8.37 ± 1.23	8.51 ± 1.19
2	8.64 ± 1.16	8.66 ± 1.04	8.39 ± 1.19	8.57 ± 1.12
3	8.58 ± 1.17	8.44 ± 1.10	8.40 ± 1.24	8.48 ± 1.15
4	8.23 ± 1.21	8.22 ± 1.21	8.23 ± 1.17	8.23 ± 1.18
5	7.96 ± 1.32	7.80 ± 1.31	8.02 ± 1.25	7.99 ± 1.28
6	7.60 ± 1.46	7.72 ± 1.38	7.77 ± 1.44	7.70 ± 1.40
7	7.49 ± 1.36	7.60 ± 1.50	7.82 ± 1.34	7.64 ± 1.39
8	7.36 ± 1.49	7.27 ± 1.47	7.46 ± 1.34	7.36 ± 1.41
9	7.07±1.43	7.32 ± 1.45	7.67 ± 1.39	7.35 ± 1.42
10	7.13 ± 1.33	7.26 ± 1.55	7.66 ± 1.37	7.35 ± 1.41

Data in w/kg mean ± std. dev.

**Table 3:** Mean subject performance for average velocity.

Bench Press Set	Supplement	Caffeine	Placebo	Total
1	0.48 ± 0.09	0.49 ± 0.08	0.49 ± 0.08	0.49 ± 0.08
2	0.46 ± 0.10	0.49 ± 0.10	0.47 ± 0.08	0.47 ± 0.09
3	0.47 ± 0.09	0.47 ± 0.10	0.47 ± 0.08	0.47 ± 0.09
4	0.45 ± 0.09	0.46 ± 0.10	0.47 ± 0.08	0.46 ± 0.09
5	0.45 ± 0.10	0.46 ± 0.10	0.47 ± 0.08	0.46 ± 0.09
6	0.44 ± 0.09	0.45± 0.10	0.46 ± 0.09	0.45 ± 0.09
7	0.43 ± 0.09	0.44 ± 0.10	0.47 ± 0.11	0.44 ± 0.10
8	0.43 ± 0.10	0.44 ± 0.12	0.47 ± 0.10	0.44 ± 0.11
9	0.41 ± 0.10	0.43 ± 0.11	0.46 ± 0.09	0.43 ± 0.10
10	0.41 ± 0.09	0.45 ± 0.12	0.47 ± 0.09	0.44 ± 0.10

All data in m/s mean ± std. dev.
